# Adjunctive Intravenous Magnesium Sulfate for Postoperative Pain and Opioid Reduction in Lower Extremity Orthopedic Surgery: A Double-Blind Randomized Controlled Trial

**DOI:** 10.3390/jcm15052055

**Published:** 2026-03-08

**Authors:** Alvian Reza Muhammad, Raden Besthadi Sukmono, Aida Rosita Tantri, Elvan Wiyarta

**Affiliations:** 1 Department of Anesthesiology and Intensive Therapy, Faculty of Medicine, University of Indonesia-Dr. Cipto Mangunkusumo National General Hospital, Jakarta 10430, Indonesia; besth25@gmail.com (R.B.S.); aidatantri@yahoo.com (A.R.T.); 2 Intensive Care Department, University of Indonesia Hospital, Depok 16424, Indonesia; elvan.wiyarta@ui.ac.id

**Keywords:** magnesium sulfate, postoperative pain, opioid reduction, orthopedic surgery, randomized controlled trial

## Abstract

**Background/Objectives:** Postoperative pain in lower extremity orthopedic surgery remains inadequately controlled. Magnesium sulfate may serve as an effective adjunct to reduce pain and opioid use. To evaluate the efficacy and safety of intravenous magnesium sulfate (30 mg/kg) as an adjuvant to ketorolac. **Methods:** Randomized, double-blind, placebo-controlled, parallel-group superiority trial. Sixty adult patients undergoing elective lower limb orthopedic surgery were randomized (1:1) to receive either intravenous magnesium sulfate (30 mg/kg) or placebo over 60 min before surgery. All patients received standard anesthesia and postoperative ketorolac with morphine PCA. The primary outcomes were postoperative pain (VAS) and morphine consumption over 24 h. Secondary outcomes included time to first analgesic request, serum magnesium levels, and adverse events. Allocation was concealed via opaque envelopes, and blinding was maintained for participants, clinicians, assessors, and analysts. **Results:** All 60 patients completed the trial. The magnesium group showed significantly lower VAS scores and reduced 24 h morphine use (median 6 mg vs. 8 mg, *p* < 0.001), with longer time to first analgesic request (540 vs. 300 min, *p* < 0.001). Four patients (13%) in the magnesium group had transient hypotension; no serious adverse events occurred. **Conclusions:** Low-dose intravenous magnesium sulfate safely reduced pain and opioid needs in orthopedic surgery and may be considered in multimodal analgesia strategies.

## 1. Introduction

Postoperative pain following orthopedic procedures—particularly those involving the lower extremities—is among the most intense and challenging to manage effectively [1,2]. Despite established protocols and the routine use of nonsteroidal anti-inflammatory drugs (NSAIDs), evidence suggests that a substantial proportion of patients continue to experience moderate to severe pain postoperatively [3,4]. A multicenter survey reported that up to 80% of surgical patients suffer from inadequate postoperative pain relief, with orthopedic procedures ranked among the highest in pain intensity [5].

This persistent inadequacy underscores the need for improved multimodal analgesic strategies. One promising pharmacologic adjunct is magnesium sulfate (MgSO_4_), which exerts analgesic effects through multiple mechanisms including antagonism of N-Methyl-D-Aspartate (NMDA) receptors, calcium channel blockade, and stabilization of neuronal membranes [6,7,8]. These actions help attenuate central sensitization and reduce neuronal hyperexcitability, both of which contribute to postoperative pain [6,7,8]. Additionally, magnesium has demonstrated anti-inflammatory properties, further reinforcing its potential as an adjuvant analgesic [9].

Several randomized controlled trials have demonstrated the analgesic efficacy of intravenous magnesium sulfate across various surgical settings. In a study of patients undergoing hysterectomy, Taheri et al. found that preoperative administration of 50 mg/kg of magnesium sulfate significantly reduced pain scores at 6, 12, and 24 h postoperatively [6]. Similar findings were reported in breast surgery and orthopedic populations, with reduced opioid consumption and prolonged time to first analgesic request [10]. Recent evidence also demonstrates that perioperative magnesium infusion may contribute to reduced intraoperative blood loss and improved multimodal analgesia outcomes in spine surgery settings, further supporting its pleiotropic perioperative effects [11]. Major lower extremity orthopedic procedures, including joint replacement, open reduction and internal fixation, and reconstructive surgeries, are associated with substantial postoperative nociceptive input and frequently require significant opioid administration during the early recovery period. Effective multimodal analgesia is therefore particularly critical in this population to facilitate early mobilization, functional rehabilitation, and reduction in opioid-related adverse effects. Despite this clinical importance, data specifically evaluating moderate-dose intravenous magnesium sulfate in lower limb orthopedic surgery remain limited.

However, most studies have used relatively high doses of magnesium (e.g., 50 mg/kg), which may increase the risk of adverse events such as hypotension or bradycardia [8,12]. The safety and efficacy profile of lower-dose regimens, such as 30 mg/kg, remains underexplored—particularly in the context of major orthopedic surgery. In the present study, 30 mg/kg was selected as a moderate-dose strategy intended to preserve the analgesic benefits of magnesium while reducing the likelihood of dose-related adverse hemodynamic effects, thereby enhancing its practical applicability in routine orthopedic perioperative care.

This randomized, double-blind, placebo-controlled trial aimed to evaluate the efficacy and safety of intravenous magnesium sulfate at 30 mg/kg as an adjuvant to ketorolac for postoperative pain control and opioid-sparing effects in patients undergoing lower extremity orthopedic surgery. We hypothesized that intravenous magnesium sulfate at a dose of 30 mg/kg, administered prior to surgery, would significantly reduce postoperative pain intensity and 24 h morphine consumption compared with placebo when used as part of a multimodal analgesia regimen in lower extremity orthopedic surgery.

## 2. Methods

### 2.1. Study Design and Setting

This was a prospective, randomized, double-blind, placebo-controlled clinical trial conducted at Cipto Mangunkusumo National General Hospital, Jakarta, Indonesia—a tertiary referral center with high orthopedic surgical volume. The study was designed with a parallel-group structure and a 1:1 allocation ratio to test the superiority of intravenous magnesium sulfate versus placebo as an adjunct to ketorolac in reducing postoperative pain and opioid consumption in patients undergoing lower extremity orthopedic surgery. Given the expected variability in postoperative pain profiles across different orthopedic procedures, randomization was stratified according to surgical category to ensure balanced distribution of procedure types between groups. This design approach was intended to minimize potential confounding related to differences in surgical invasiveness and baseline nociceptive burden.

The study protocol was reviewed and approved by the Health Research Ethics Committee of the Faculty of Medicine, Universitas Indonesia–Cipto Mangunkusumo National General Hospital, Jakarta, Indonesia (approval code KET-879/UN2.F1/ETIK/PPM/.00.02/2022; approval date 29 August 2022). Written informed consent was obtained from all participants prior to enrollment. The trial was registered in a public clinical trials registry at ClinicalTrials.gov (identifier NCT05609955; last update 11 February 2022; record verification November 2022). This manuscript adheres to the Consolidated Standards of Reporting Trials (CONSORT) 2010 guidelines [13], including all essential items outlined in the checklist for randomized controlled trials (see Table S1). 

### 2.2. Participants

Patients were screened consecutively from surgical lists between October and December 2022. Eligible participants were adults aged 18 to 60 years scheduled for elective lower limb orthopedic procedures under general anesthesia. Inclusion criteria required patients to have an American Society of Anesthesiologists (ASA) physical status classification of I to III and the ability to provide informed consent [14]. Exclusion criteria included unstable hemodynamics, impaired renal function (defined as serum creatinine >1.2 mg/dL), neuromuscular disorders, a history of bronchial asthma, and known hypersensitivity to ketorolac or morphine.

All participants were provided with detailed information about the study and gave written informed consent prior to enrollment. Patients or members of the public were not involved in the trial design, conduct, data analysis, or dissemination of the findings.

### 2.3. Randomization, Allocation Concealment, and Blinding

Randomization was performed using a computer-generated sequence created with permuted block randomization (block size = 4), stratified by type of orthopedic procedure. The sequence was prepared in advance by an independent research assistant with no role in patient enrollment or outcome assessment. Allocation was concealed using sequentially numbered, opaque, sealed envelopes (SNOSE) managed by the hospital pharmacy [15].

The pharmacy team was responsible for preparing and labeling the study medications as “research drug.” Both the magnesium sulfate and placebo solutions were identical in volume, appearance, and packaging, ensuring blinding was maintained. Participants, anesthesiologists, outcome assessors, intraoperative staff, and data analysts were all blinded to the group allocation throughout the study. No breaches of blinding or emergency unblinding occurred.

### 2.4. Interventions

In the preoperative holding area, patients randomized to the intervention group received intravenous magnesium sulfate at a dose of 30 mg/kg body weight, diluted in 100 mL of 0.9% sodium chloride. The placebo group received 100 mL of 0.9% sodium chloride alone. Both solutions were infused via a peripheral vein over 60 min, ending just before the patient was transferred to the operating room.

General anesthesia was induced with fentanyl (2 µg/kg), propofol (2 mg/kg), and either atracurium (0.5 mg/kg) or rocuronium (0.6 mg/kg) as a neuromuscular blocking agent. Endotracheal intubation was performed using appropriately sized tubes (7.0 mm internal diameter for females, 7.5 mm for males). Anesthesia was maintained with sevoflurane (1.5–2.0 vol%) in 50% oxygen and air. Supplemental fentanyl boluses (0.5–1 µg/kg) were administered intraoperatively if heart rate or mean arterial pressure increased by ≥20% from baseline values in the absence of other identifiable causes, ensuring standardized analgesic titration across participants.

At the end of surgery, neuromuscular blockade was reversed using neostigmine (0.04 mg/kg) and atropine (0.02 mg/kg). All patients received intravenous ketorolac 30 mg immediately postoperatively, followed by 30 mg every 8 h thereafter. Postoperative analgesia was supported by intravenous patient-controlled analgesia (PCA) with morphine. PCA settings were standardized: 1 mg per demand, a 5 min lockout interval, and a maximum limit of 6 mg/h. Prior to surgery, all patients received standardized education on PCA use delivered by trained anesthesia personnel. The instruction included a structured verbal explanation and device demonstration covering the purpose of PCA, appropriate indications for activation (e.g., moderate to severe pain), the lockout interval mechanism, maximum dose limits, and safety principles emphasizing that only the patient should press the demand button. Patients were given the opportunity to ask questions to ensure adequate understanding before surgery. No other opioids or sedatives were administered during the first 24 h unless required for rescue analgesia.

If signs of hypermagnesemia (e.g., bradycardia, hypotonia, respiratory depression) were observed, patients were to be withdrawn from the study and treated with intravenous calcium gluconate per protocol. However, no such events occurred during the trial.

### 2.5. Outcome Measures

The primary outcomes were postoperative pain intensity and opioid consumption. Pain intensity was assessed using the Visual Analog Scale (VAS) ranging from 0 to 10, recorded both at rest and during movement at four time points: 0, 6, 12, and 24 h after surgery [16]. Opioid consumption was quantified as the total morphine dose administered through the PCA device in the first 24 h postoperatively.

Secondary outcomes included the time to first request for morphine via PCA, serum magnesium levels before and after intervention, and the incidence of adverse events. Adverse events of interest were hypotension (defined as systolic blood pressure < 90 mmHg) [17], nausea, vomiting, heartburn, muscle weakness, and cardiac arrhythmia. Pain assessments were conducted by trained members of the Acute Pain Service (APS) who were blinded to treatment allocation [18]. All assessments were performed only after the patient achieved an Aldrete score of at least 9 and could respond to simple verbal commands. Blood samples for magnesium level assessment were taken via peripheral venipuncture before administration and one hour after the infusion concluded.

### 2.6. Harms Assessment

Adverse events were systematically monitored and documented during and after surgery up to 24 h postoperatively. The severity, timing, and clinical response were recorded by the APS team. Hypotension was managed using intravenous ephedrine boluses. No participants exhibited signs or symptoms of clinically significant hypermagnesemia, and all serum magnesium concentrations remained within physiologic limits. Four patients in the intervention group experienced transient post-induction hypotension, which was promptly treated and resolved without further complications. No serious adverse events or study withdrawals occurred.

### 2.7. Sample Size

Sample size was calculated based on the assumption of a 20% reduction in 24 h postoperative morphine consumption as the clinically meaningful difference between groups. Assuming a standard deviation of 3 mg, a two-tailed alpha level of 0.05, and a statistical power of 80%, a minimum of 27 participants per group was required. To account for potential dropouts or exclusions, 30 participants were recruited per group. Sample size estimation was performed using G*Power version 3.1 (Heinrich-Heine-Universität Düsseldorf, Germany) [19].

### 2.8. Statistical Analysis

Statistical analyses were performed using R Version 4.3.0 (R Foundation for Statistical Computing, Vienna, Austria) [20]. The Kolmogorov–Smirnov test was used to evaluate data distribution. Normally distributed variables were expressed as means with standard deviations and analyzed using independent *t*-tests. Non-normally distributed data were reported as medians with ranges and analyzed using the Mann–Whitney U test. Categorical variables were presented as counts and percentages and compared using Chi-square or Fisher’s exact test, as appropriate.

All analyses were conducted according to the intention-to-treat principle, including all randomized participants in the groups to which they were assigned. There were no missing data or protocol deviations requiring exclusion or imputation. Randomization was expected to ensure baseline comparability; therefore, no additional adjustment for potential confounders was performed. Statistical significance was defined as *p* < 0.05. No interim, subgroup, or sensitivity analyses were conducted, and all analyses were prespecified in the study protocol. The statistical analyses were conducted according to the pre-specified analysis plan. Between-group differences are presented as absolute mean differences with corresponding *p*-values, allowing direct clinical interpretation of treatment effects in terms of pain scores and opioid consumption.

## 3. Results

### 3.1. Participant Flow and Recruitment

**Figure 1:**
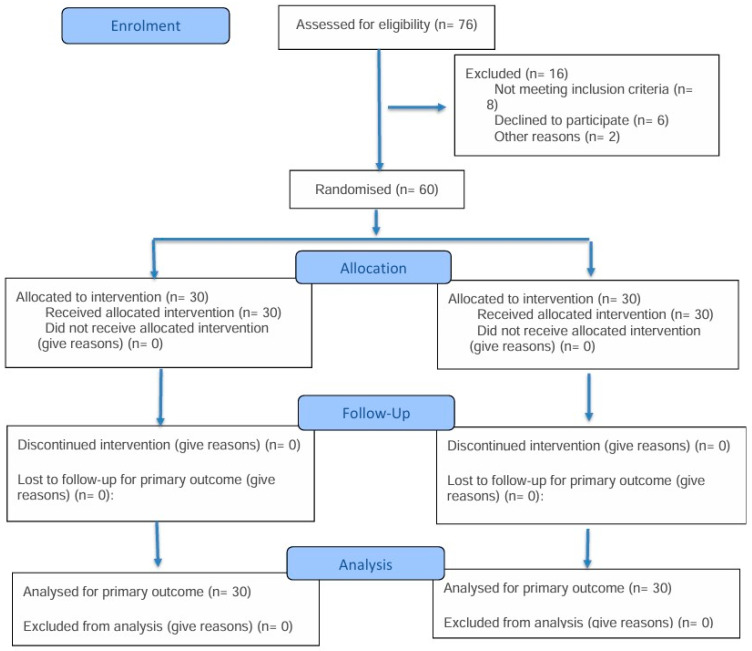
Flow diagram of participant screening, randomization, allocation, follow-up, and analysis.

Between 1 October and 31 December 2022, a total of 76 patients were screened for eligibility. Of these, 16 were excluded prior to randomization: 8 did not meet inclusion criteria, 6 declined to participate, and 2 were excluded for administrative reasons. Sixty eligible participants were randomized equally into the magnesium sulfate group (*n* = 30) and the placebo group (*n* = 30). All participants received the allocated interventions, completed the 24 h follow-up period, and were included in the final analysis without loss to follow-up or protocol deviation (Figure 1).

### 3.2. Baseline Characteristics

Baseline demographic and clinical characteristics were comparable between the two groups (Table 1). Median age was 33 years in the placebo group and 27 years in the magnesium group. Both groups were balanced in terms of gender, ASA classification, anthropometric measurements, and distribution of surgical procedures. The median duration of surgery was slightly longer in the magnesium group (210 min) than in the placebo group (198 min). Intraoperative fentanyl usage was similar across groups.

### 3.3. Pain Intensity

**Figure 2:**
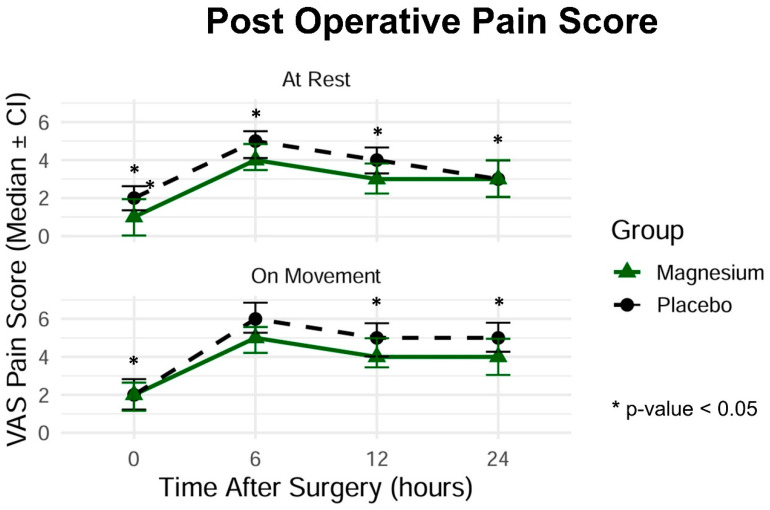
Postoperative pain scores at rest and during movement at 0, 6, 12, and 24 h, measured using the Visual Analog Scale (VAS). Data presented as medians (min–max) and corresponding *p*-values.

Postoperative pain scores measured using the Visual Analog Scale (VAS) at rest and during movement are presented in Figure 2. At rest, pain intensity was significantly lower in the magnesium group at 0, 6, 12, and 24 h postoperatively (all *p* < 0.05). During movement, statistically significant reductions in pain scores were observed at 0, 12, and 24 h; however, the difference at 6 h was not significant (*p* = 0.075). These results support the analgesic efficacy of intravenous magnesium sulfate as an adjunct to ketorolac.

### 3.4. Opioid Requirements

The magnesium group had significantly reduced morphine consumption in the first 24 postoperative hours, with a median of 6 mg (range: 0–12), compared to 8 mg (range: 0–14) in the placebo group (*p* < 0.001; Table 2). Additionally, the time to first analgesic request was longer in the magnesium group (median 540 min) than in the placebo group (median 300 min), indicating a longer duration of pain control (*p* < 0.001).

### 3.5. Safety and Adverse Events

No serious adverse events were recorded (Table 3). Four patients (13%) in the magnesium group developed transient hypotension after anesthetic induction, which was effectively managed with vasopressors. No patients in either group experienced nausea, vomiting, heartburn, muscle weakness, or cardiac arrhythmia. No withdrawals due to adverse events occurred.

### 3.6. Serum Magnesium Levels

Serum magnesium concentrations increased slightly in the magnesium group following the infusion (Table 4). Pre-intervention levels were 2.3 mg/dL (range: 1.9–3.3), increasing to 2.4 mg/dL (range: 2.0–3.2) post-infusion, though this change was not statistically significant (*p* = 0.623). No participant showed evidence of hypermagnesemia.

### 3.7. Analysis Population

All 60 randomized patients were included in the analyses of both primary and secondary outcomes. No missing data or exclusions occurred. All analyses were conducted according to the intention-to-treat principle, and all were prespecified in the trial protocol. No interim, subgroup, or sensitivity analyses were performed.

## 4. Discussion

This randomized, double-blind, placebo-controlled trial showed that intravenous magnesium sulfate at a dose of 30 mg/kg administered before surgery, as an adjunct to ketorolac, resulted in significantly lower postoperative pain scores and reduced opioid requirements in patients undergoing lower extremity orthopedic surgery. Compared to placebo, patients receiving magnesium reported lower VAS scores both at rest and during movement across the first 24 h, and they required significantly less morphine. Importantly, the observed reductions in pain scores and opioid consumption should be interpreted not only in terms of statistical significance but also clinical relevance. In the context of acute postoperative pain, even modest reductions in VAS scores and opioid requirements may translate into improved patient comfort, reduced opioid-related adverse effects, and facilitation of early mobilization. Therefore, the magnitude of absolute differences observed in this study supports potential clinical benefit beyond statistical findings alone. The time to first analgesic request was also markedly prolonged, indicating more sustained analgesic effect. These findings support the role of low-dose magnesium sulfate as an effective opioid-sparing strategy within a multimodal analgesia regimen.

The results of this study are consistent with and extend previous findings in the literature. Several clinical trials and meta-analyses have demonstrated that intravenous magnesium sulfate can enhance postoperative analgesia by reducing pain intensity and opioid use. Taheri et al. observed significant reductions in pain scores and opioid requirements with 50 mg/kg magnesium sulfate in patients undergoing abdominal surgery [6]. Similarly, Shin et al. demonstrated analgesic benefit in orthopedic populations, particularly in patients undergoing total knee arthroplasty [10]. A meta-analysis by Avci et al. further confirmed that systemic perioperative magnesium reduces pain and morphine consumption in various surgical contexts [21]. However, these studies predominantly used higher doses (typically 50 mg/kg), raising concerns about dose-dependent side effects such as hypotension, bradycardia, or flushing [12]. Our study adds novel evidence showing that a lower dose of 30 mg/kg still provides clinically meaningful analgesia with a more favorable safety profile.

Magnesium sulfate is believed to exert its analgesic effects through antagonism of N-Methyl-D-Aspartate (NMDA) receptors and voltage-gated calcium channels [22]. By blocking NMDA receptors, magnesium inhibits the excitatory glutamatergic pathways responsible for central sensitization, a key mechanism underlying postoperative and chronic pain [22,23,24]. In addition to its central action, magnesium has been shown to possess anti-inflammatory properties, modulating cytokine production and reducing neuronal hyperexcitability [9,25,26,27]. These mechanisms may explain the observed analgesic and opioid-sparing effects even at a lower dose.

In our study, serum magnesium levels increased modestly after infusion but remained within normal physiological limits, with no cases of symptomatic hypermagnesemia. Four patients (13%) in the magnesium group experienced transient hypotension following induction of anesthesia, which resolved promptly with low-dose ephedrine and did not necessitate discontinuation of the study drug. Importantly, no patients experienced muscle weakness, arrhythmia, or significant gastrointestinal symptoms such as nausea, vomiting, or heartburn. This safety profile aligns with the findings of earlier studies that suggest most adverse effects occur at higher doses or with rapid infusions [12]. However, it should be noted that the present study was not powered to detect rare or uncommon adverse events. Although no serious complications were observed, the relatively modest sample size limits definitive conclusions regarding safety, and larger studies are required to more comprehensively characterize the risk profile of perioperative magnesium administration.

This study has important clinical implications. In the context of the ongoing opioid crisis, interventions that safely reduce opioid consumption are highly valuable. The ability of magnesium sulfate to reduce opioid requirements while maintaining effective pain control makes it a promising adjunct in enhanced recovery after surgery (ERAS) protocols, particularly in orthopedic populations where postoperative pain is often intense and prolonged. Furthermore, given its low cost, ease of administration, and favorable safety profile, magnesium sulfate may be especially valuable in low- and middle-income country (LMIC) settings, where access to advanced analgesic modalities is limited.

Despite these strengths, our study has several limitations. First, it was conducted at a single tertiary referral hospital, which may limit the generalizability of findings to other settings or surgical populations. Second, although we stratified randomization by surgical procedure, the study still included a heterogeneous mix of orthopedic operations—ranging from arthroscopy to joint replacement—which may have introduced variability in pain experiences and analgesic needs. Although stratified randomization was implemented to balance procedure categories between groups, we did not perform procedure-specific subgroup analyses. It is therefore possible that the magnitude of treatment effect may vary across different types of orthopedic surgery. Future studies focusing on more homogeneous surgical populations or adequately powered subgroup analyses would help clarify whether the analgesic benefit of magnesium differs according to surgical invasiveness. This heterogeneity, while reflective of real-world surgical practice, may have reduced internal precision. Third, pain intensity was assessed using the VAS, a unidimensional measure. While VAS is widely used, it may not fully capture other relevant aspects of the pain experience such as functional limitation, emotional distress, or opioid-related side effects.

Additionally, the study focused only on short-term outcomes within the first 24 h postoperatively. While this window is clinically relevant for immediate recovery and pain control, future studies should examine whether the benefits of magnesium sulfate persist beyond 24 h, affect rehabilitation milestones, or reduce the risk of chronic post-surgical pain. Lastly, although our sample size was sufficient to detect differences in pain and opioid use, it may not have been powered to detect rare adverse events or subgroup-specific effects. Future adequately powered multicenter trials with longer follow-up periods are warranted to confirm these findings and to evaluate longer-term outcomes, including functional recovery and the potential prevention of chronic postoperative pain.

Nonetheless, the strengths of the trial—including its randomized design, strict blinding procedures, high protocol adherence, and complete follow-up—support the robustness of the findings. This study contributes valuable new evidence that even at a reduced dose, intravenous magnesium sulfate can meaningfully improve postoperative pain outcomes in orthopedic surgery patients. Given its accessibility, affordability, and opioid-sparing potential, magnesium sulfate deserves consideration as a standard component of perioperative multimodal analgesia, particularly in resource-limited settings.

## 5. Conclusions

In this randomized, double-blind controlled trial, intravenous magnesium sulfate at a dose of 30 mg/kg administered prior to lower extremity orthopedic surgery significantly reduced postoperative pain intensity and morphine consumption when used as an adjunct to ketorolac. The intervention demonstrated sustained analgesic effects, prolonged time to first opioid demand, and was associated with a favorable safety profile, with only minor and transient hemodynamic effects. These findings support the use of low-dose magnesium sulfate as a safe, effective, and accessible component of multimodal analgesia in orthopedic surgical patients.

## Figures and Tables

**Table 1:** Baseline demographic and clinical characteristics of study participants.

Characteristic	Placebo Group (*n* = 30)	Magnesium Group (*n* = 30)
Age (years)	33 (18–60)	27 (18–60)
Sex		
Male	19 (52.8%)	17 (54.2%)
Female	11 (47.2%)	13 (45.8%)
ASA		
I	9 (30%)	9 (30%)
II	21 (70%)	21 (70%)
Body weight (kg)	62.65 ± 13.59	63.61 ± 12.50
Height (cm)	161.06 ± 6.61	162.45 ± 7.65
Type of surgery		
Hip replacement	5 (16%)	4 (13%)
Hip arthroplasty	2 (6%)	3 (3%)
Knee replacement	6 (20%)	10 (33%)
ORIF	8 (26%)	6 (20%)
Arthroscopy	6 (20%)	4 (13%)
Other	3 (3%)	3 (3%)
Duration of surgery (minutes)	198 (45–440)	210 (45–480)
Intraoperative fentanyl (μg)	300 (100–750)	225 (100–500)

ASA: American Society of Anesthesiologists; kg: kilogram; cm: centimeter; ORIF: Open Reduction and Internal Fixation; μg: microgram; min: minutes.

**Table 2:** Total morphine consumption in the first 24 h and time to first analgesic request, with group comparisons and *p*-values.

Variable	Placebo Group (*n* = 30)	Magnesium Group (*n* = 30)	*p*-Value
Total morphine consumption (mg)	8 (0–14)	6 (0–12)	<0.001
Time to first PCA request (minutes)	300 (240–600)	540 (300–780)	<0.001

*n*: number of participants; mg: milligrams; PCA: patient-controlled analgesia.

**Table 3:** Adverse events observed in both groups.

Adverse Event	Placebo Group (*n* = 30)	Magnesium Group (*n* = 30)
Post-induction hypotension	0 (0%)	4 (13%)
Nausea and/or vomiting	0 (0%)	0 (0%)
Heartburn	0 (0%)	0 (0%)
Muscle weakness	0 (0%)	0 (0%)
Cardiac arrhythmia	0 (0%)	0 (0%)

*n*: number of participants; %: percent.

**Table 4:** Serum magnesium levels in the magnesium group before and after intervention.

Time Point	Serum Magnesium (mg/dL)	*p*-Value
Before intervention	2.3 (1.9–3.3)	0.623
After intervention	2.4 (2.0–3.2)	

mg/dL: milligrams per deciliter.

## Data Availability

All data generated or analyzed during the study are included in this published article.

## Supplementary Materials

The following supporting information can be downloaded at: https://www.mdpi.com/article/10.3390/jcm15052055/s1, Table S1: CONSORT 2010 checklist of information to include when reporting a randomised trial [13].
